# The Use of “Ferrum Candens” in Diseased Joints

**Published:** 1872-08

**Authors:** A. W. Calhoun

**Affiliations:** Vienna


### FOREIGN CORRESPONDENCE.

### THE USE OF "FERRUM CANDENS" IN DISEASED JOINTS.

BY A. W. CALHOUN, M. D.

Every physician has had more or less to do with diseases of different characters involving the various joints of the upper and lower extremities; and probably the experience of each and all will confirm the assertion that such, in general, may be classed amongst the most inflexible of all, affecting the body, and that, as a rule, their treatment is productive of less good fruit. Even when progressing favorably, the cure of all inflammations of the joints, more especially when chronic, resulting from whatever cause, is accomplished only after the careful and continuous management of a series of months or even years, which naturally enough, is exhausting to the patience both of the sufferer and surgeon; and the latter not unfrequently finds himself at that unpleasant point where he is more than willing to surrender his case into the hands of any professional brother who can afford relief.

In the course of the last six or eight months, I have had the opportunity of witnessing the treatment of a large number of diseased joints in various hospitals and clinics, and of following the progress of most of the cases from beginning to end; and I beg leave to report the results of my observations and a few of the cases.

Of course, in these affections, as in all others, cases are brought into hospitals suffering from every stage of the disease, and with their diseases depending upon almost every imaginable cause; in short, cases presenting usually an unfavorable prognosis, resulting from long delay, bad nutrition, improper management, and a multiplicity of other causes. As a commencement with each individual, the milder remedies are brought into use, such as rest, bandaging, counter-irritants of various kinds, &c.—which in an occasional case is sufficient to check the disease; but in the majority of instances, after a reasonable trial, all of these are cast aside, as possessing in themselves no essential good, and other more severe and more deeply impressing means or applications brought forward. The favorite and well deserving remedy is "Ferrum Candens," followed by the confinement of the limb in a plaster of Paris bandage. The results of this treatment strongly recommend it; for where the disease is not already beyond recall, its working is most flattering, and, in comparison to other methods, rapid.

As is well known, scrofulous and phthisical patients are the most frequent subjects of joint troubles—the seed of the disease being sown, as it were, when the sufferers are spoken into life. In fact, experience has taught us, that even when no constitutional symptoms have previously manifested themselves, the appearance of such affections should always excite suspicion and investigation, since they with constitutional ills so often go hand in hand. This portion of Europe is no exception to this rule, for, according to my observation, two-thirds or three-fourths of the long list of cases of joint affections, belong to the above class of constitutional maladies, and at first glance present the opposite of a good prognosis, under whatever treatment. Yet even amongst these, the influence af the "hot iron" and the auxiliary management, works most satisfactorily upon the local troubles, and brings to a cure numbers of these diseases that had formerly withstood a multitude of means, in the hands of the most skilled and experienced, and is now adopted as a standard remedy in all the hospitals of Berlin, and fast growing in favor with the surgeons of this city, Vienna.

By no means is it intended to lay before your readers this particular treatment of inflammation and other joint affections, as a new idea—as something hitherto unknown or untried; for just the opposite is a well known fact, viz.: that it was in existence, but improperly practiced by our predecessors far in the past, and been handed down to the present generation to be made practically useful. Like all other remedies of similar severe nature, it has from its introduction had its stout opponents, and has many times fallen and been again raised by some appreciative hand—each time taking a firmer foothold in the affections of the profession. To a few bright medical lights of our own age belong the honor of having brought it into such use, and for such diseases as to make it, so to speak, a boon to the unfortunate, and promising a rich harvest for the future. Specially prominent amongst these, are Profs. Langenbeck, of Berlin, and Billroth, of Vienna—the latter giving also a special place to "Galvano Caustic," which, to a great extent, is "Ferrum Candens" in a modified form.

The diseases in which this has been particularly used (not exclusively, however), have been inflammations in the various joints, secondary to a constitutional taints of the system, dropsical swellings, and inflammations resulting from and continuing after direct injury.

Below I give a few cases illustrating the mode of procedure, and rapidity of the good effect, which shows itself in a short time, when at all to be attained by this treatment. Under each case is given also, in a few words, the entire after treatment, &c.

*Dropsy of the Knee Joint.*—Woman, aged 40, of quite a delicate constitution, and mother of several children; has been confined to her room for several months from a dropsical inflammation of the right knee joint, and having been treated since its first appearance, in numberless ways, both constitutionally and locally, without effect, in her own house, is to-day, February 19th, 1872, brought into hospital. The joint is immensely enlarged, showing much fluid upon palpation; feverish and painful to the touch. All other means having been thoroughly tested, Prof. Langenbeck decided to make immediate use of the "actual cautery." Patient is chloroformed, and the iron, heated to a "white heat," applied above and below the knee, and on either side, leaving free the popliteal space. The cautery is used to the extent of burning through and completely charring the entire epidermis, with which it comes in contact, as this is thought requisite to produce a sufficient amount of external inflammation to relieve the joint itself, and bring about absorption. The limb is slightly flexed and enveloped in a wet roller, or bandage, from about midway between foot and knee to midway between knee and hip joint, and still over this wadding and the bandage of plaster of Paris. Patient is put at absolute rest in bed, and no internal medicines given, aside from opiates. At the end of ten or twelve days, this bandage was removed, the parts cleansed from the slight suppuration of the wounds, and a new one of similar character applied. After the expiration of a like interval, this second plaster bandage was also removed, and the limb found mostly healed of the burns, the swelling reduced, and the joint but slightly sensitive upon pressure. She suffered but little pain, and almost no fever, during this treatment of three or four weeks. From this time on the common cloth bandage was used, and patient kept quiet for several weeks, when she was dismissed—all of her previously annoying and dangerous symptoms having disappeared, and only a moderate amount of stiffness remaining.

It is probably well to make mention, that when applying the plaster of Paris bandage, the leg must be slightly flexed at the knee, as the pain following the laying on of the bandage upon a perfectly extended limb, is apt to lead one to suppose it to be altogether too tight, and suggest its removal, when in fact the pain is entirely due to the inconvenient position of the leg.

Sometimes it happens, but not usually, that the accumulation of pus resulting from the burns, causes the removal of the bandage, and its subsequent removal. More generally the suppuration is not so very profuse, soon drying underneath the bandage, and doing no harm. This point must of course be looked after by the surgeon, and managed as the case demands.

Two other cases of dropsical knee joint were in like manner treated, a few days after the one aboved noted, and as the nature of the affection in each instance was so similar (with the exception that in the two last, the sensitiveness of the joints was much less) and the treatment so exactly the same as in the first, it is unnecessary to give any further report of their management or after progress. In one of the last the knee was punctured by the Trocar, and the fluid drawn away, and a small quantity of tincture iodine injected. The cautery and plaster of Paris bandage were then used as described in the first case. These cases also recovered in a short time, without any evil symptoms whatever attending their progress to cure.

*Chronic Inflammation of Wrist Joint.*—Man aged 25. Several months ago, without any apparent cause, the joint became very painful to the touch, and has continued to increase in size and sensitiveness, until at present, March 2nd, 1872, the wrist is a large, angry-looking, swollen and painful mass, upon which, in the past few months, the virtue of such applications as irritating fluids and ointments, and all manner of bandaging, has been exhausted, without benefit. All of these are now dispensed with, as unsuited to the case, and after chloroforming the patient, the "Ferrum Candens" is applied over the whole of the posterior portion of the joint, burning entirely through the skin, and, as in the former cases, leaving it in a charred condition. Covering the parts with wadding and a flannel roller, immediately placed on the plaster of Paris bandage, including not only the wrist itself, but the hand and entire fore-arm, as this insures absolute rest of every muscle exerting any influence upon the joint. On March 23d the bandages were removed, when the burns were found slightly ulcerated, but presenting a healthy appearance. During these three weeks patient has suffered not the least inconvenience from weight of bandages, &c. (taking daily his usual exercise), and the previous continual pain has diminished to a dull, heavy, unpleasant sensation in the parts, which can reasonably be attributed to the burns themselves. Dressing the wounds with simple ointment, applied the common roller bandage, and will, if necessary, re-apply in a few days the plaster of Paris.

March 29th—In the last few days has improved rapidly in all respects—wounds having nearly healed, and the pain entirely disappearing.

Two weeks after this, patient presented himself, having no pain, the parts nearly of their normal size, and no undue sensitiveness. Is directed to wear a loose bandage around his wrist, and his arm in a sling, and to give his hand rest for a few weeks longer. As he made his appearance at the clinic no more after this time, it is reasonable to suppose a perfect cure followed.

*Inflammation of Knee Joint* (*Osteo Porosa*).—Man aged 26.—

When 14 years of age, (now 13 years ago), patient fell, and so injured his knee, that severe inflammation ensued; but by long continued treatment, the joint became once more free. One or two years ago he again fell, injuring the same knee, which having since then greatly enlarged, and being continually inflamed, is a source of great trouble and pain—compelling his almost constant confinement to his room or his bed. The nature of the swelling and inflammation constitute that affection known as "Osteo Porosa." The gradual contraction of the muscles has drawn the limb very near to a right angle, and partial anchylosis has also resulted.

Chloroform is administered and the "Ferrum Candens" applied March 13th, 1872, on each side of the knee, above and below, leaving the epidermis over the patella and in the popliteal entire. A simple wet bandage is placed over the entire extremity, the patient put to bed, and by the use of heavy weights, the leg gradually straightened to a position in which it can be made hereafter useful. This occupied several days, at the end of which time the plaster of Paris bandage is used, and allowed to remain three or four weeks, when afterwards the ordinary bandages were substituted.

The parts improved rapidly—the pain and inflammation disappearing altogether, and the swelling to some extent—the patient suffering in the meantime but little, as a consequence of this apparently severe treatment. Of course anchylosis and a certain amount of enlargement of the joint will continue; but the chief point has been accomplished, viz: the dissipation of the inflammation and pain, which had so long made the patient's life a burden to him. In this condition—that is, with the constant pain and chronically inflamed state of the joints, of several years' duration, removed, he is dismissed a few weeks later, with the limb, although anchylosed, in an infinitely better condition and position for future usefulness.

At all events, this remedy, in the hand of the prudent, is a source of no evil—on the contrary, its timely and cautious use, not unfrequently influences wonderful and rapid changes for the better—and to those who may have been unsuccessful in similar diseases, with other means, this is suggested as possessing properties and qualities well worthy of trial.

*Vienna, June 15th,* 1872.

